# Clinical Audit of Postoperative Documentation in the Kassala Teaching Hospital, Sudan: Compliance With Royal College of Surgeons of England Guidelines

**DOI:** 10.7759/cureus.90948

**Published:** 2025-08-25

**Authors:** Alaa M. A. Adam, Ibrahim Adil Hamadelniel Alhadi, Mohamed Almustafa Magdi M Mohamed Ahmed, Mohamed Abdelrahman, Monair Yousif Abdelgader Alnaeem, Raneem Alsiddig Ali Ibrahim, HeifAljenan MohammedAhmed Yousif Mohammed, Mustafa Hussein Mohammed Mustafa, Tasneem Sulieman Mohamed Tahameed, Marwa Waleed Abdulrahman Mohammed, Mohamed Alghazali, Shima Abdalraheem Elnadeef Hussein, Mohammed Osman Ahmed Osman, Ahmed Mohammed Abdalganey Alhaj, Mohammed Maher Abdallah Awadelkareem, Mogahid Hamdan Adam Ahmed, Abdarheem Mohamed Ibrahim Mohamed, Rodina Noaman Ahmed Mohammed, Ahmed Abdelgabar Sheikh Idries Almadani, Elwathig Abdalla

**Affiliations:** 1 General Surgery, Kassala Teaching Hospital, Kassala, SDN; 2 General Surgery, Royal College of Surgeons of Edinburgh, Edinburgh, GBR; 3 Human Anatomy, University of Gezira, Wad Madani, SDN; 4 General Surgery, University of Gezira, Wad Madani, SDN; 5 Surgery, Elrazi University, Cairo, EGY; 6 General Surgery, Al Neelain University, Khartoum, SDN; 7 Urology, University Hospitals of Morecambe Bay, Kendal, GBR; 8 General Physician, National Ribat University, Khartoum, SDN; 9 Trauma and Orthopaedics Surgery, Ibrahim Malik Teaching Hospital, Khartoum, SDN; 10 Internal Medicine, The National Ribat University, Khartoum, SDN; 11 Trauma and Orthopaedics, Our Lady of Lourdes Hospital, Drogheda, IRL; 12 General Surgery, University of Kordofan, El Obeid, SDN; 13 Dentistry, University of Medical Sciences and Technology, Khartoum, SDN; 14 Orthopaedic Surgery, Managil Teaching Hospital, Managil, SDN

**Keywords:** clinical audit, postoperative documentation, rcs guidelines compliance, standardized templates, targeted interventions

## Abstract

Background

Accurate postoperative documentation is critical for patient safety, continuity of care, and medico-legal protection. This audit assessed postoperative documentation at the Kassala Teaching Hospital, Sudan and its compliance with the Royal College of Surgeons of England (RCS) guidelines, and evaluated the impact of targeted interventions.

Methods

A retrospective pre-intervention audit reviewed 44 postoperative notes from April to May 2025. A root cause analysis identified deficiencies (e.g., lack of awareness, no standardized templates, time constraints). Interventions included staff education, standardized templates, visual reminders, and enhanced supervision. A post-intervention audit of 51 notes evaluated improvements.

Results

Pre-intervention compliance was high for operative procedure (88.6%), findings (88.6%), and postoperative instructions (93.2%), but critically low for deep vein thrombosis (DVT) prophylaxis (25.0%), prosthesis identification (40.9%), and surgeon’s signature (45.5%). Post-intervention, significant improvements occurred in operative diagnosis (from 45.5% to 86.4%; +89.8%) and date/time documentation (from 65.9% to 88.6%; +34.5%). However, DVT prophylaxis (27.3%), prosthesis identification (43.2%), and surgeon’s signature (47.7%) remained suboptimal.

Conclusion

Targeted interventions significantly improved overall compliance with RCS documentation standards. Persistent gaps in documenting DVT prophylaxis, prostheses, and signatures highlight systemic challenges. Sustained improvement requires institutional changes (e.g., electronic templates, mandatory tracking systems, regular audits) and ongoing staff training. Re-auditing is recommended to ensure long-term adherence.

## Introduction

Accurate and comprehensive postoperative documentation is a cornerstone of safe and effective surgical care. Operative and postoperative notes are critical not only for ensuring continuity of care and facilitating multidisciplinary communication, but also for medico-legal protection, clinical audit, and quality improvement initiatives [1,2].

Recognizing these imperatives, the Royal College of Surgeons of England (RCS) has developed and periodically updated the Good Surgical Practice guidelines, which establish evidence-based standards for surgical documentation [1,2]. These guidelines specify the essential components of operative and postoperative records, including patient identification, date and time, surgical team members, diagnosis, procedure details, intraoperative findings, complications, and postoperative instructions [1-5].

Despite the existence of such comprehensive guidance, multiple studies across diverse healthcare settings have demonstrated persistent deficiencies in the quality and completeness of surgical documentation [3-5]. Audits conducted in tertiary hospitals in Pakistan, India, and the United Kingdom have consistently reported suboptimal adherence to RCS guidelines, with frequent omissions of key data elements in the operative notes [3-5]. However, these studies also highlight that targeted interventions, such as education, feedback, and standardized templates, can lead to significant improvements in compliance [3-5].

In low- and middle-income countries, including Sudan, the quality of surgical documentation has received increasing attention. Recent audits in Sudanese teaching hospitals have revealed similar challenges, with operative notes often lacking critical information required by international standards [6-10]. These deficiencies may compromise patient safety, hinder clinical decision-making, and limit the utility of records for audit and research purposes [10-11]. Nevertheless, there is a paucity of published data specifically addressing compliance with RCS guidelines in the Kassala Teaching Hospital, a major referral center in eastern Sudan.

The present clinical audit seeks to systematically evaluate the compliance of postoperative documentation with the RCS guidelines in the Kassala Teaching Hospital, Sudan. By identifying current practices and specific areas for improvement, this study aims to inform targeted interventions that will enhance the quality and safety of surgical care in this setting.

## Materials and methods

Pre-intervention phase

A retrospective review of 44 postoperative notes from the Kassala Teaching Hospital (April 10 to May 5, 2025) assessed compliance with the RCS documentation guidelines. Each note was evaluated for key parameters such as patient identification, surgical team names, operative details, complications, and postoperative instructions.

The review found high compliance in documenting the operative procedure, diagnosis, and postoperative care. However, significant gaps were identified in recording estimated blood loss, deep vein thrombosis (DVT) prophylaxis, identification of prostheses/materials used, and the surgeon’s signature. Other areas with suboptimal compliance included the anesthetist’s name and wound closure method. These findings highlighted the need for targeted interventions to improve documentation practices and ensure alignment with international standards.

Root cause examination

The root causes were identified by directly interviewing surgeons and doctors through a structured root cause examination process. No formal scales were used; rather, qualitative insights from these interviews highlighted the key contributing factors. These included: (1) Lack of awareness: Some surgical staff were unaware of the full extent of RCS documentation requirements; (2) Absence of standardized templates: The operative note format varied, leading to the omission of certain parameters; (3) Time constraints: High workload and time pressures often resulted in rushed or incomplete documentation; (4) Limited feedback: There was no systematic review or feedback process for operative notes, so errors and omissions were not routinely addressed; (5) Inconsistent training: New staff and trainees did not consistently receive orientation on documentation standards.

Intervention phase

During the intervention phase, several targeted actions were implemented at the Kassala Teaching Hospital to improve compliance with RCS documentation guidelines. Surgical staff received educational sessions on proper operative note-taking, and standardized documentation templates were introduced to ensure all required parameters were included. Visual reminders such as posters and checklists (Appendix) were displayed in key areas, and feedback from the initial audit was shared with the surgical teams to highlight areas needing improvement. Senior staff also increased supervision and support for junior doctors, promoting adherence to best practices in documentation.

Post-intervention phase

In the post-intervention phase, 51 postoperative notes from the Kassala Teaching Hospital were reviewed to assess improvements following targeted interventions. These notes represented different patients undergoing a similar range of surgical procedures and were handled by the usual surgical staff. The sample size was determined using total coverage sampling for the post-intervention period, providing sufficient data to observe trends in documentation improvements while remaining feasible for detailed review.

The audit showed significant increases in compliance, particularly in documenting operative diagnosis (from 45.5% to 86.4%) and date/time of surgery (from 65.9% to 88.6%). Moderate improvements were also noted in incision type, tissue details, and DVT prophylaxis documentation. Despite these gains, some areas such as DVT prophylaxis, prostheses identification, and surgeon’s signature remained below optimal levels. Overall, the interventions led to measurable enhancements in documentation quality, though ongoing efforts are needed to achieve full compliance.

Data analysis

The analysis of data was facilitated by Microsoft Excel 2016 (Microsoft Corporation, Redmond, WA), utilizing descriptive statistics such as frequencies and percentages to summarize compliance rates. A comparative analysis was also undertaken to discern patterns and prevalent shortcomings in documentation practices, thereby highlighting opportunities for enhancement.

## Results

A total of 44 postoperative notes were reviewed in the first audit cycle and 51 notes in the second cycle following the intervention. Overall, there was an improvement in compliance with the RCS postoperative documentation guidelines across most parameters.

The most significant improvement was observed in the documentation of the operative diagnosis, which increased from 45.5% in the first cycle to 86.4% in the second cycle, representing an 89.8% relative improvement. Documentation of date and time also showed a marked increase from 65.9% to 88.6% (34.5% improvement). There were moderate improvements in the documentation of incision type (from 65.9% to 70.5%, 6.9% improvement), details of tissue removed, added or altered (from 63.6% to 68.2%, 7.2% improvement), and DVT prophylaxis (from 25.0% to 27.3%, 9.1% improvement).

Other parameters demonstrated smaller gains, including identification of the operator and assistant (from 84.1% to 88.6%, 5.4% improvement), name of anesthetist (from 63.6% to 65.9%, 3.6% improvement), and documentation of closure technique (from 59.1% to 61.4%, 3.8% improvement). The recording of extra procedures and identification of prostheses/material used both improved from 40.9% to 43.2% (5.6% improvement each).

Several parameters, such as type of operation, operative procedure carried out, operative findings, prophylactic antibiotics, and postoperative care instructions, were already at high levels of compliance (>85%) in the first cycle and showed only marginal improvements in the second cycle.

Despite these improvements, certain areas such as DVT prophylaxis (27.3% in the second cycle), identification of prostheses/material used (43.2%), and surgeon’s signature (47.7%) remain below optimal levels and require further attention.

Table 1 compares documentation compliance between the first (n=44) and second (n=51) audit cycles across key operative parameters, and highlights percentage improvements, with notable gains in areas such as operative diagnosis, date and time, and personnel identification.

**Table 1:** Summary of documentation compliance rates for selected operative parameters across the two audit cycles Data representation: Values are shown as N (%); Values represent the number and percentage of cases with complete entries, with the final column indicating the percentage improvement from the first to the second cycle; Statistical test used: Chi-square test for categorical comparisons; Significance thresholds: Statistical significance was considered at p<0.05 (*), and p<0.001 (**); Test statistic: Chi-square (χ²) values are reported alongside p-values to enhance interpretability.

Parameter	First cycle (n=44)	Second cycle (n=51)	Percentage improvement	Test statistic	p-value
Date and time	29 (65.9%)	39 (88.6%)	34.50%	χ² = 6.93	p=0.008**
Type of operation	38 (86.4%)	39 (88.6%)	2.60%	χ² = 0.10	p=0.75
Name of operator and assistant	37 (84.1%)	39 (88.6%)	5.40%	χ² = 0.36	p=0.55
Name of anesthetist	28 (63.6%)	29 (65.9%)	3.60%	χ² = 0.06	p=0.80
Operative procedure carried out	39 (88.6%)	40 (90.9%)	2.60%	χ² = 0.13	p=0.72
Incision type	29 (65.9%)	31 (70.5%)	6.90%	χ² = 0.29	p=0.59
Operative diagnosis	20 (45.5%)	38 (86.4%)	89.80%	χ² = 17.36	p<0.001**
Operative findings	39 (88.6%)	40 (90.9%)	2.60%	χ² = 0.13	p=0.72
Problems/Complications	20 (45.5%)	21 (47.7%)	4.90%	χ² = 0.05	p=0.82
Extra procedures and rationale	18 (40.9%)	19 (43.2%)	5.60%	χ² = 0.05	p=0.82
Tissue details	28 (63.6%)	30 (68.2%)	7.20%	χ² = 0.24	p=0.62
Prostheses/Material used	18 (40.9%)	19 (43.2%)	5.60%	χ² = 0.05	p=0.82
Closure technique	26 (59.1%)	27 (61.4%)	3.80%	χ² = 0.06	p=0.80
Anticipated blood loss	20 (45.5%)	21 (47.7%)	4.90%	χ² = 0.05	p=0.82
Prophylaxis antibiotics	39 (88.6%)	40 (90.9%)	2.60%	χ² = 0.13	p=0.72
DVT prophylaxis	11 (25.0%)	12 (27.3%)	9.10%	χ² = 0.07	p=0.79
Postoperative care instructions	41 (93.2%)	42 (95.5%)	2.40%	χ² = 0.17	p=0.68
Surgeon’s signature	20 (45.5%)	21 (47.7%)	4.90%	χ² = 0.05	p=0.82

In summary, the implementation of targeted interventions resulted in measurable improvements in the quality of postoperative documentation at the Kassala Teaching Hospital. However, ongoing efforts are needed to achieve full compliance with RCS guidelines, particularly in areas with persistently low documentation rates.

## Discussion

This audit demonstrated that structured interventions significantly enhanced compliance with RCS's postoperative documentation standards [1,2] at the Kassala Teaching Hospital. The most substantial improvements were observed in operative diagnosis documentation (45.5% to 86.4%) and surgery date/time recording (65.9% to 88.6%). These findings align with studies from Pakistan and India, where educational interventions and standardized templates similarly improved documentation completeness [3,4]. The observed gains likely resulted from multifaceted strategies including staff education, template standardization, and visual reminders, approaches consistent with successful quality improvement initiatives in comparable low-resource settings [5,6].

Despite overall progress, critical deficiencies persisted. Documentation of DVT prophylaxis showed minimal improvement (25.0% to 27.3%), while identification of prostheses/materials (43.2%) and surgeon signatures (47.7%) remained suboptimal. These specific gaps mirror challenges identified in Sudanese hospitals where systemic constraints, such as inconsistent material tracking systems and workflow pressures, hinder compliance [6,7]. The persistently low recording of DVT prophylaxis may reflect inadequate institutional protocols for venous thromboembolism risk assessment or prioritization of acute intraoperative elements over preventive measures.

Root cause analysis revealed several contributing factors, such as a lack of awareness of RCS standards among staff [1,2], the absence of standardized documentation templates, time constraints during clinical duties, and inconsistent training for new personnel. These findings corroborate audits from Dongola and Port Sudan Teaching Hospitals, where similar systemic barriers were identified [5,6]. The absence of structured feedback mechanisms further perpetuated documentation omissions, as noted in audits from Bangladesh and the UK [4,8].

Incomplete documentation poses tangible patient safety risks. Omission of DVT prophylaxis documentation could lead to thromboembolic events [1,2], while undocumented prostheses complicate future surgical revisions. These gaps also impair clinical audit trails and medico-legal protection [1,2]. 

To address persistent gaps, the following measures are recommended: integration of standardized templates into electronic medical records where feasible; implementation of mandatory prosthesis tracking systems; automated clinical decision support for DVT risk assessment; and establishment of weekly random note audits with feedback to surgical teams [3-4]. Sustainability requires continuous education programs, particularly for new staff and trainees. Re-auditing in six to 12 months is essential to evaluate long-term compliance and assess correlations between documentation completeness and patient outcomes [5,7].

Despite the observed improvements, this study has several limitations. The audit was conducted in a single institution, limiting generalizability across other hospitals with differing workflows or resources. The absence of electronic health records constrained the scope of interventions, and documentation assessments relied on manual chart reviews, which may be subject to reviewer bias. Additionally, the short post-intervention follow-up period precludes an assessment of long-term sustainability.

## Conclusions

This audit highlighted that targeted interventions such as education sessions, standardized templates, and enhanced supervision significantly improved compliance with the RCS postoperative documentation guidelines at the Kassala Teaching Hospital. Notable progress was seen in areas like operative diagnosis and recording of date and time, demonstrating the effectiveness of simple, low-cost strategies. However, persistent shortcomings in documenting DVT prophylaxis, prosthesis identification, and surgeon signatures continue to pose challenges. These gaps emphasize the need for institutional reforms, including electronic templates, mandatory tracking systems, and structured feedback mechanisms. Sustained improvement will depend on continuous staff training, regular audits, and embedding documentation standards into routine surgical practice to safeguard patient safety and medico-legal accountability.

## Figures and Tables

**Figure 1:**
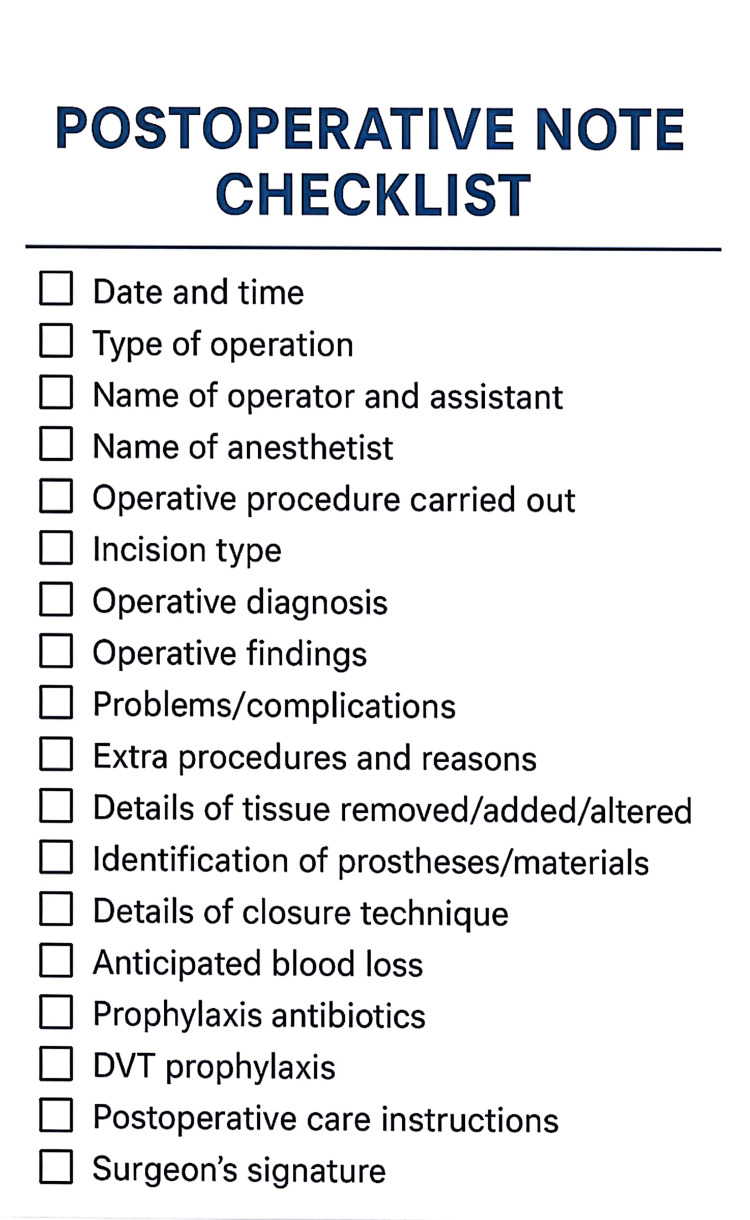
Checklist poster used in the interventions
